# A297 EXAMINING THE IMPACT OF INFLAMMATORY BOWEL DISEASE IN PRIMARY SCLEROSING CHOLANGITIS PATIENTS POST LIVER TRANSPLANTATION

**DOI:** 10.1093/jcag/gwad061.297

**Published:** 2024-02-14

**Authors:** G Malhi, L A Diaz, G Punchhi, R Mortuza, M Khan, M Brahmania, V Jairath, J Arab

**Affiliations:** Gastroenterology, Western University, London, ON, Canada; Pontificia Universidad Catolica de Chile Facultad de Medicina, Santiago, Chile; Gastroenterology, Western University, London, ON, Canada; Gastroenterology, Western University, London, ON, Canada; Gastroenterology, Western University, London, ON, Canada; University of Calgary Cumming School of Medicine, Calgary, AB, Canada; Gastroenterology, Western University, London, ON, Canada; Gastroenterology, Western University, London, ON, Canada

## Abstract

**Background:**

Primary sclerosing cholangitis (PSC) is an immune-mediated disease that is characterized by biliary inflammation and fibrosis. It is associated with inflammatory bowel disease (IBD) in 80% of cases. To date, the impact of IBD in liver transplantation (LT) recipients is not completely understood.

**Aims:**

To assess the impact of IBD in individuals with PSC who underwent liver transplantation (LT) in terms of graft survival, infections, and mortality.

**Methods:**

This was a retrospective cohort study that included individuals with PSC who received a LT between 1999–2021. Statistical analysis included Kaplan-Meier survival curves, a binary logistic regression to estimate infection risk, and a competing-risk analysis to estimate post-LT mortality (with re-transplantation being a competing risk).

**Results:**

122 LT recipients were included. Mean age at LT was 44.9±12.6 years old. The median MELD-Na at LT was 22 [17–28]. Twenty-nine (23.8%) LT recipients died during follow-up (median 1,248 [413–3,857] days) and 5 (4.1%) required re-transplantation (median 1,460 [923–2,563] days). Estimated graft survival was 93.2% (95%CI: 86.9%–96.6%) at 1 year and 81.3% (95%CI: 72.5%–87.6%) at 5 years. An adjusted competing-risk model demonstrated that increasing age (sHR 1.05, 95%CI: 1.01–1.10; p=0.018), baseline MELD-Na (sHR 1.07, 95%CI: 1.02–1.12; p=0.005), and ERCP requirements before LT (sHR 6.33; 95%CI: 1.63–24.65; p=0.008) were associated with higher post-LT mortality, while IBD was not associated with post-LT mortality (sHR 1.02, 95%CI: 0.38–2.70; p=0.962). The incidence of infections after LT was 50.8% at 30 days. IBD was not associated with development of infections at 30 days (OR 1.01, 95%CI: 0.98–1.04; p=0.615) post-LT.

**Conclusions:**

Based on this retrospective review, an older age, higher MELD-Na at LT and prior ERCP requirements were independently associated with a higher mortality post-LT in PSC patients. However, a background history of IBD was not associated with higher mortality or with infections post-LT.

Table 1: Baseline Demographics

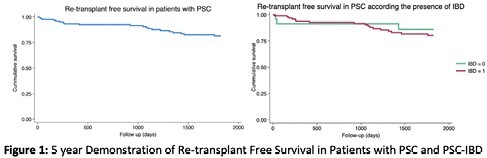

**Funding Agencies:**

None

**Table:** 

Category	Number (%)
**PSC**	122 (100)
Classic PSC	104 (85.3)
PSC-AIH Overlap	17 (13.9)
Small Duct PSC	1 (0.8)
**Age**	
ampersand:003C30	17 (13.9)
30-50	59 (48.4)
ampersand:003E50	46 (37.7)
**Sex**	
Male	91 (74.6)
Female	31 (25.4)
**IBD History**	
IBD Present	99 (81.2)
No IBD	23 (18.8)

